# Economic Evaluation of Population-Level Chronic Kidney Disease Interventions in the UK National Health Service

**DOI:** 10.36469/001c.134075

**Published:** 2025-04-30

**Authors:** George Agathangelou, Matthew Graham-Brown, Aisling C McMahon, George Xydopoulos, Larisa Gofman, Jacob Jaffe

**Affiliations:** 1 Health Economics and Outcomes Research Team ZS Associates, London, UK; 2 Department of Cardiovascular Sciences University of Leicester, UK; 3 Kidney Research UK, Peterborough, UK; 4 HEOR ZS Associates

**Keywords:** chronic kidney disease, cost-utility analysis, SGLT-2 inhibitors, economic burden, population-level Markov model, National Health Service

## Abstract

**Background:** Chronic kidney disease (CKD) affects 13% of the global population, is predicted to be the fifth leading cause of premature death by 2040, and is associated with increased risk of cardiovascular disease and acute cardiovascular events. With an aging population and rising diabetes rates, the prevalence of CKD is expected to escalate in the United Kingdom, leading to substantial healthcare costs. When patients reach end-stage kidney disease, interventions such as dialysis and transplantation are required. Dialysis is not only extremely costly but is also associated with a diminished quality of life and significantly elevated mortality. **Objectives:** This study assesses the cost-effectiveness of several population-level interventions designed to manage CKD, including its progression to end-stage kidney disease. **Methods:** A population-level Markov model was developed to evaluate the cost-effectiveness and population health impacts of 4 key interventions, individually and combined: (1) early/improved diagnosis, (2) enhanced CKD management, (3) increased use of SGLT-2 inhibitors, and (4) higher rates of pre-emptive live donor transplantation. The model incorporates both NHS direct costs and broader economic impacts, with a 10-year horizon and quarterly cycles. Two scenarios were analyzed: a base case (based on disease progression probabilities) and a constrained case (where growth in the number of patients receiving dialysis and transplantation is limited to historical rates observed in the UK National Health Service). **Results:** All interventions demonstrated cost-effectiveness, with the combined approach preventing 10 351 deaths and yielding 48 381 quality-adjusted life-years (QALYs) at a cost of £7675 per QALY in the base case scenario. In the constrained scenario, the combined interventions demonstrated cost-effectiveness, preventing 10 026 deaths and yielding 47 514 QALYs at a cost of £22 767 per QALY. **Conclusions:** The results demonstrate the cost-effectiveness of population level interventions for management of CKD, and the significant burden of dialysis, with avoidance of progression to dialysis a key driver of QALY gains and cost offsets.

## BACKGROUND

Chronic kidney disease (CKD) is a progressive condition defined by a sustained decline in kidney function over a minimum period of 3 months, irrespective of its underlying causes.^1^ It is a global health concern affecting 13% of the global population and is predicted to be the fifth leading cause of premature death by 2040.^2^ In 2019, there were approximately 69.7 million cases of CKD globally, with an average annual increase of 1.19% since 1990,^3^ while a new modeling analysis by AstraZeneca, the IMPACT CKD study, forecasts up to 16.5% of the population across 8 countries will suffer from CKD by 2032,^4^ primarily due to population growth, aging, and rising rates of diabetes and uncontrolled hypertension^2,5^ Notably, early stages of CKD are often asymptomatic or exhibit nonspecific symptoms, meaning diagnoses are often made incidentally during routine health checks.

Past studies estimated that CKD cost England’s National Health Service (NHS) approximately £1.45 billion per year in 2010, equivalent to about 1.3% of the total NHS spending.^6^ More than half of this cost is attributable to kidney replacement therapies such as dialysis (hemodialysis and peritoneal dialysis) and kidney transplantation, both of which are resource-intensive from financial and health resource perspectives.^6^ Another challenge within the United Kingdom is the underdiagnosis and delayed diagnosis among Black and South Asian communities and in areas of high deprivation.^7^ Patients classified as having end-stage kidney disease (ESKD), where renal function has deteriorated significantly, require kidney replacement therapies for survival. While most patients with CKD do not progress to ESKD, they are at elevated risk for cardiovascular events, such as stroke, myocardial infarction, heart failure, and early cardiovascular mortality.^8^

Hemodialysis is burdensome for patients and healthcare systems. The therapy is typically delivered at a hospital or outpatient dialysis center, although home hemodialysis is possible for some. Patients on hemodialysis typically require at least 3 sessions per week, lasting around 4 hours per session. Peritoneal dialysis is a home-based treatment that requires the patient or caregivers to perform multiple treatments per day.^9^

Patients receiving dialysis have a mortality risk from cardiovascular disease (CVD) that is up to 20 times greater than that of the general population and are at increased risk for cognitive decline and dementia.^10^ The financial implications are significant, with an approximate annual cost per patient of around £34 000, excluding costs related to transportation, which may add another £8000 annually.^11^

Kidney transplantation generally leads to superior health outcomes, including improved life expectancy, compared with remaining on dialysis.^12^ However, challenges persist regarding donor availability, with average waiting times for a deceased donor transplantation ranging from 2 to 3 years.^13^

Strategies to prevent reaching end-stage kidney disease (ESKD) have focused on controlling hypertension, particularly with the use of renal-angiotensin system antagonists such as angiotensin-converting enzyme inhibitors (ACE-i) and angiotensin II receptor blockers (ARBs), as recommended by UK National Institute for Health and Care Excellence guidelines.^14^ Numerous randomized controlled trials and meta-analyses have demonstrated the cardio-renal protective effects of ACE-i/ARBs.^15^ Despite these benefits, there remains a notable underutilization of ACE-i/ARBs in clinical practice, with only approximately 53% of eligible CKD patients receiving these medications.^11^ More recently, sodium-glucose transport protein 2 (SGLT-2) inhibitors have emerged as a promising therapeutic option for CKD management. A recent network meta-analysis by Chen et al indicated that SGLT-2 inhibitors significantly reduced cardiovascular mortality and heart failure incidents by 25% (risk ratio [RR]=0.75, respectively) in CKD patients.^16^ These benefits were observed even when patients were well managed on ACE-i/ARB therapy. Additionally, SGLT-2 inhibitors were associated with a reduction in kidney-specific events.^16^

Given the increasing prevalence of CKD within the population, it is crucial for the UK NHS to explore cost-effective interventions to prevent both the progression to ESKD and associated excess cardiovascular events related to CKD among these patients. This study aims to evaluate the cost-utility of 4 distinct interventions applied individually or in combination throughout different stages of the CKD treatment pathway, comparing them against current standard care practices.

## METHODS

### Study Design

This study employed a population-based Markov model to evaluate the economic burden of CKD under current practice over a 10-year time horizon and the cost-effectiveness of 4 established interventions for CKD within the context of the NHS. The study was undertaken from the perspective of the UK NHS, in line with National Institute for Health and Care Excellence (NICE) health technology assessment guidelines (NICE PG 36).

The starting position of the model was the distribution of patients by disease state as of 2023 based on the best available data in literature. This was projected forward at a population level for 10 years based on the transition probabilities included within the model, including an incidence of new CKD patients from the broader UK population.

For the base case scenario, it should be noted that this forecast implicitly assumes that any demand for healthcare would be met, and it is not constrained by current system capacity (ie, forecast of patients in dialysis is based on the number of patients requiring hemodialysis).

### Model Structure

The model design was informed by a comprehensive literature review and qualitative insights from leading renal academics and clinicians in the United Kingdom as part of a broader piece of research on kidney disease.^17–19^ Several potential model structures were identified in the literature on modeling CKD, which included Markov models, microsimulation, and combinations of multiple model types.^19^ A population-based Markov model was ultimately chosen due to the multiple decision problems and availability of data from recent economic evaluations that relied on Markov models.^11,20^

This model diverges from traditional CKD models that typically concentrate on ESKD by encompassing all stages of CKD as per the global “Kidney Disease: Improving Global Outcome” (KDIGO) guidelines.

**Figure 1.**
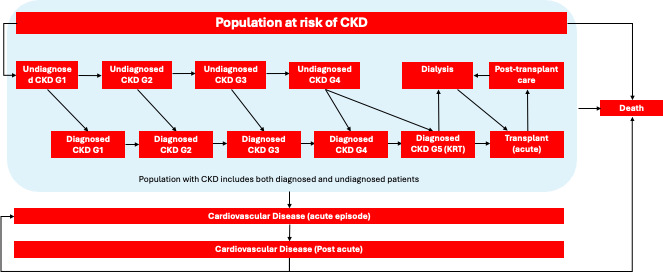
Model Structure Abbreviation: CKD, chronic kidney disease.

The Markov model consisted of 16 disease states (**Figure 1**), with each cycle representing a duration of 3 months and a total time horizon of 10 years. The target population for this cost-effectiveness analysis was any patient with CKD. Given that the prevalence of CKD is estimated to be around 10% across all adults in England, the UK adult population was considered the at-risk group for developing CKD in the model.^21^ Patients could progress through CKD stages from undiagnosed stage 1 to stage 5, with transitions influenced by incidence rates derived from current epidemiological data.

In the model, patients flow from a general at-risk group (UK adult population) into a stage 1 undiagnosed CKD state. They then progress through CKD stages, either remaining undiagnosed or becoming diagnosed, with the possibility of experiencing a cardiovascular event or kidney transplantation.

The model accounted for both direct healthcare costs incurred by the NHS and patient outcomes measured in quality-adjusted life-years (QALYs), applying a standard discount rate of 3.5% for both costs and outcomes.

### Scenario Analysis: Evaluating 4 Interventions

**Base case scenario:** A baseline forecast of the economic burden of CKD was produced for the base case scenario (no interventions applied). The following interventions were then applied to identify their impact in mitigating the economic burden of CKD (key parameters included in **Supplementary Table S1**):

*Intervention 1: Early/improved diagnosis of CKD*. This intervention targets early diagnosis of CKD in underserved populations through outreach and selective screening. Modeling involved estimating the undiagnosed stage 1, 2, and 3 prevalent population from Black, Asian, and Minority Ethnic (BAME) communities and adjusting transition probabilities between undiagnosed and diagnosed states, resulting in a 25% reduction in the undiagnosed BAME population over the time horizon of the model.

*Intervention 2: Enhanced CKD management through better hypertension control*. This intervention targets eligible patients with CKD who are either untreated or not receiving standard care according to clinical guidelines (eg, adequate blood pressure management). Current evidence suggests that up to 36% of patients with diagnosed CKD who should be receiving ACEi/ARBs are not. This intervention models the impact of closing this gap.^12^ This intervention was applied by adjusting the transition probability from stage 3 to stage 4 CKD for diagnosed patients.

*Intervention 3: Increased utilization of SGLT-2 inhibitors.* This intervention aims to estimate the impact of increased uptake of new medications such as SGLT-2 inhibitors, which have clinical evidence to show a reduction in cardiovascular events and slowing progression to ESKD. As the baseline data used for the model predated NICE approval for SGLT-2 inhibitors, it was assumed that 0% of eligible patients were receiving SGLT-2 inhibitors at model initiation (t_0_). Model transition probabilities from diagnosed stage 4 CKD to stage 5 CKD and cardiovascular acute episode were adjusted to reflect the impact of an increase to 100% of the eligible population (18.8% of the stage 4 CKD prevalent population) receiving SGLT-2 inhibitors over the time horizon of the model.

*Intervention 4: Higher rates of pre-emptive live donor kidney transplantation*. This intervention models increased rates of kidney transplantation. NHS clinicians identified this as a potential solution, which has been achieved in some areas through increased awareness campaigns to patients entering ESKD and their families, at an annual cost of £149 000 per year for outreach, resulting in a 100% increase in the number of pre-emptive transplants. This intervention was applied by changing the transition probability between stage 5 CKD and transplantation.

**Constrained scenario:** As avoidance of hemodialysis was expected to be a significant driver of cost and QALY benefits within the model, given its significant burden, a further scenario was produced wherein the growth in hemodialysis and transplantation activity was constrained to historic growth rates through adjustment of the associated transition probabilities as a means on sensitizing the model to this variable.

### Transition Probabilities

Transition probabilities between CKD states were extracted from relevant literature, ensuring that they represented CKD progression and related cardiovascular events.^8,12,22–28^ Where annual transition probabilities were reported, they were mathematically transformed to quarterly transition probabilities to align with the model cycle duration.

This was achieved by transforming the annual probability (*P*_annual_) to rate and then the rate (*r*) to a quarterly probability (*P*_quarterly_) using the following formulas:

$$r=-ln(1-P_{annual})$$

$$P_{quarterly}=1-e^{-r_{quarterly}}$$

$$r_{quarterly}=r/4$$

The probability of death associated with each disease stage was calculated based on UK life tables and the relative risk of death by CKD stage.^8,24^ Other transition probabilities in the model were not age dependent.

Additionally, it was assumed that patients with undiagnosed CKD progress through the stages at a higher rate due to a lower likelihood of receiving treatment compared with diagnosed patients.^29^

Baseline transition probabilities were adjusted to reflect the impact of interventions and create the constrained scenario. **Supplementary Tables S2-S5** show the transition probabilities for the baseline scenario without and with interventions, and the constrained scenario without and with interventions, respectively.

At model initiation (t_0_), the UK adult population of 53 million people was categorized into different disease states **(Supplementary Table S6),** including an at-risk group that does not have CKD, based on estimates from published literature.^12,21,27^ The distribution by CKD stage was informed by the Health Survey for England 2016, which represents one of the largest random national samples of the UK adult population (n = 3464). This survey involved single measurements of estimated glomerular filtration rate and urine albumin-to-creatinine ratio.

The proportion of patients undiagnosed in CKD stages 1 to 4 was derived from a population study conducted in Oxfordshire, focusing on individuals aged 60 years and older, which found that 44% of CKD patients were undiagnosed.^27^

No patients were included in the transplantation (acute) or CVD (acute) states at the model’s initiation, as these are considered tunnel states, and no patients were assigned to the CVD (post-acute) state due to a lack of available data on the prevalence of this population.

### Economic Evaluation

Costs and QALYs were assigned to each disease state in the model, and for each intervention, such that the interventions could be compared individually and collectively against a base case of standard care. Only costs associated with CKD were considered, and, as such, no costs were assigned to the at-risk group. As CKD is generally asymptomatic before stage 3, the same QALY value was assigned to the at-risk group as the stage 1 and stage 2 CKD groups. Sensitivity analyses were conducted to assess the robustness of the results.

### Resource Use and Costs

The analysis considered only the direct healthcare costs relevant to a UK payer perspective. In the model, each health state was allocated a quarterly direct medical cost based on published literature (**Table 1**). Healthcare costs related to CKD or acute CVD events with prior history of CKD were used. Costs from prior years were inflated to 2022 costs using the consumer price inflation (CPI) for health from the Office of National Statistics **(Supplementary Table S7)**.^29^ Costs for undiagnosed CKD stages 1 and 2 were assumed to be negligible, given that patients typically do not exhibit symptoms related to CKD in these stages and these patients would not have been treated by a healthcare provider for CKD. For undiagnosed CKD stages 3 and 4, it was assumed that they would have similar levels of healthcare costs related to their CKD as diagnosed patients, despite these patients not being diagnosed. As costs sourced from literature for CKD stages 1 through 5 only included hospital costs, an assumption was made that an additional £60 annual cost of primary care appointments should be added to account for 2 to 3 primary care visits based on clinical input.

**Table 1.** One-Way Sensitivity Analysis

**Health State**	**Quarterly Cost (£)**	**Reference**	**Utility**	**Reference**
No kidney disease	0	Assumption	0.8534	Jesky et al^30^
Undiagnosed CKD G1	0		0.8534	
Undiagnosed CKD G2	0		0.8534	
Undiagnosed CKD G3	137.1332	Kent et al^31^ + Assumption	0.8034	
Undiagnosed CKD G4	134.3032		0.7434	
Diagnosed CKD G1	16.19	Assumption	0.8534	
Diagnosed CKD G2	16.19		0.8534	
Diagnosed CKD G3	137.1332	Kent et al^31^ + Assumption	0.8034	
Diagnosed CKD G4	134.3032		0.7434	
Diagnosed CKD G5	173.6132		0.7334	
Transplantation (acute)	17 512.2411	NICE TA 77511	0.7135	McEwan et al^32^
Transplantation (post-acute)	1328.0511		0.8435	
Dialysis	8595.1611		0.5836	Lowney et al^33^
Dialysis: Transportation to dialysis	1840.1833	Roberts et al^34^	N/A	
CVD (acute)	5153.6032	Kent et al^31^	0.66	
CVD (post-acute)	218.5832		0.73	
Death	0		0	

One-way sensitivity analysis was undertaken, measuring the impact on the ICER for the combined interventions under the base case resulting from an adjustment of 7 key variables.

### Health-Related Quality of Life

Published utilities from UK data sources for each health state were employed in the model (**Table 1**). Utilities for CKD stages 1 through 5 were derived from the Euroqol EQ-5D-3L survey in a UK pre-dialysis CKD population. Utilities for patients on dialysis or who had experienced an acute CVD event were derived from an EQ-5D-3L survey in patients on dialysis and CKD patients who had undergone an acute CVD event. It was assumed there was no difference in utility between the undiagnosed and diagnosed states due to CKD having few noticeable symptoms in early stages (G1-3), and limited evidence that diagnosis alone improves patient-related quality of life.

### Sensitivity Analysis

A univariate, or 1-way, sensitivity analysis was undertaken to evaluate how altering a single input parameter at a time affected the output results of the model, focusing on the impact of each intervention, key base line transition probabilities (transition to dialysis or transplantation) and incidence of CKD. A sensitivity of ±20% was used for all sensitivities other than intervention 4 (proactive live donor transplants), which was set at ±7% to reflect that live donor transplants are a small subsets of overall kidney transplantation numbers.

## RESULTS

In the base case, the model predicts a significant increase in burden on patients and the healthcare system from CKD: 7 192 307 of people with CKD, with 141 074 of people in dialysis in year 10, and £70.6 billion cumulative direct costs over the 10 years modeled (**Table 2**). The main driver of the economic burden is the increase in dialysis, and it should be noted that this relies on the assumption that all demand is met (patients access to dialysis is not constraint by system’s capacity).

**Table 2.** Ten-Year Patient Counts for the Base Case Scenario

**Patient Counts**	**Prevalence (Year 10)**			**Incidence (Year 10)**		**Total (Years 1-10)**
	**CKD 1-2**	**CKD 3-5**	**Dialysis**	**Transplantation**	**CVD**	**Deaths**
Baseline	3 744 580	3 447 727	141 074	11 512	192 873	7 739 759
Intervention 1	3 744 759	3 447 867	140 977	11 504	192 870	7 739 578
Intervention 2	3 744 529	3 454 155	139 057	11 341	192 719	7 736 318
Intervention 3	3 744 496	3 465 898	139 034	11 343	190 419	7 733 648
Intervention 4	3 744 572	3 446 482	139 954	11 951	192 955	7 739 151
Combined Interventions 1-4	3 744 615	3 471 254	135 878	11 593	190 340	7 729 407
Difference: Baseline and combined interventions	35	23 528	−5196	82	−2533	−10 351

Abbreviations: CKD, chronic kidney disease; CVD, cardiovascular disease.

Interventions changed the number of patients in each disease state by year 10. This table sets out the prevalent and incidence (as appropriate) populations in each disease state for the baseline, then with the interventions applied individually and collectively.

The economic evaluation of healthcare interventions over a 10-year horizon revealed that all modeled interventions were deemed cost-effective, falling below the UK NICE incremental cost-effectiveness threshold (ICER) of £20 000 to £30 000 per QALY. The combination of all 4 interventions led to an ICER of £7675/QALY and was expected to increase direct costs by £371.3 million over 10 years.

The analysis of the combined effects of all 4 interventions (**Table 3**) revealed a slight increase in QALY gains when compared with the individual contributions of each intervention. Specifically, the combination resulted in a total of 48 381 QALYs, which is higher than the cumulative QALY gains of 48 221 QALYs from each intervention assessed

separately. This additional QALY gain will be referred to as the interaction factor, and accounts for overlaps in the benefits health benefits delivered across the 4 interventions.

**Table 3.** Economic Impact of Interventions in the Base Case

**Incremental Difference vs Base Case**						
	**Baseline (Before Interventions)**	**Combined Interventions 1-4**	**Intervention 1**	**Intervention 2**	**Intervention 3**	**Intervention 4**
Direct costs	£70 664 656 235	£371 335 097	£35 026 834	£275 344 012	£110 320 648	−⁠£51 468 036
QALYs	71 681 793	48 381	1262	20 079	25 735	1145
ICER		£7675	£27 749	£13 713	£4 287	−£44 939

Abbreviations: ICER, incremental cost-effectiveness threshold; QALY, quality-adjusted life-year.

Each intervention had an incremental impact on costs and QALYs. When the 4 interventions were combined (implemented at the same time), the total change in costs and QALYs was different from the sum of the 4 interventions individually due to an interaction between them. An ICER was calculated for the interventions individually and collectively.

### Constrained View Scenario

The model’s benefits were primarily driven by preventing the progression to hemodialysis. This is particularly evident when comparing the base case results to the constrained scenario, where the growth of baseline hemodialysis was limited to historical levels. Under these conditions, the ICER for the combined interventions were notably higher at

£22 767/QALY, driven primarily by the much lower prevalence of dialysis (**Table 4**).

**Table 4.** Ten-Year Patient Counts for the Constrained Scenario

**Patient Counts**	**Prevalence (Year 10)**			**Incidence (Year 10)**	**Total (Years 1 - 10)**	
	**CKD 1-2**	**CKD 3-5**	**Dialysis**	**Transplant**	**CVD**	**Deaths**
Baseline	3 744 677	3 585 097	32 768	3501	196 677	7 744 430
Interventions 1-4	3 744 717	3 604 625	32 039	3666	193 950	7 734 404
Difference	40	19 528	-729	165	-2727	-10 026

Abbreviations: CKD, chronic kidney disease; CVD, cardiovascular disease.

This table sets out the prevalent and incidence (as appropriate) populations for the baseline, then with the collective interventions applied in the constrained view scenario.

### Sensitivity Analyses

**Figure 2.**
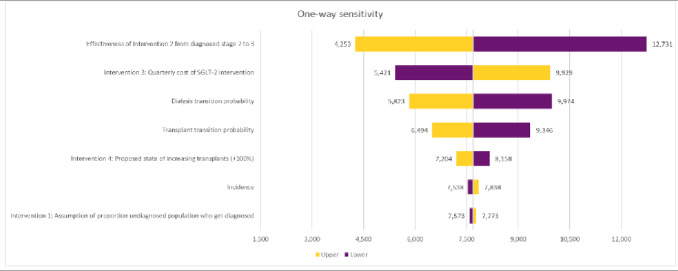
One-Way Sensitivity Analysis Abbreviations: ICER, incremental cost-effectiveness ratio; SGLT-2, sodium-glucose transport protein–2.

A univariate sensitivity analysis was conducted across the combination of all 4 interventions. Key parameters around CKD epidemiology, the impact of individual interventions, cost variables, and state utilities were included in the analysis (**Figure 2**).

One-way sensitivity analysis was undertaken, measuring the impact on the ICER for the combined interventions under the base case resulting from an adjustment of 7 key variables.

## DISCUSSION

These findings indicate that all 4 interventions are individually cost-effective, each maintaining an ICER below £30 000 per QALY. Intervention 1, earlier diagnosis, was the least cost-effective intervention, with an ICER of £27 749/QALY. Intervention 4, increased rates of preemptive live donor transplant, was cost-saving and the most cost-effective intervention at an ICER of −£44 939/QALY.

Notably, when these interventions are combined, the ICER drops to £7675 per QALY. Sensitivity analyses supported the reliability of these results across different scenarios and reveals that substantial cost savings can be realized by minimizing the duration patients spend on dialysis, which is expensive, resource-demanding, and linked to a lower quality of life.

The most significant total QALY improvements arise from enhanced CKD management through ACE inhibitors and SGLT-2 inhibitors, as they impact a larger patient demographic. Although early detection was initially less cost-effective, its value is likely to increase over time due to the lengthy progression from early CKD to ESKD. The study also points out disparities in disease progression among racial groups, underscoring the critical need for early identification, particularly in Black and South Asian populations, which face a higher risk of ESKD. While the transplantation intervention resulted in cost savings, its overall QALY gains were lower due to its narrower focus on a smaller patient group.

A key driver for the benefits shown by the model was the prevention of progression to hemodialysis. This is highlighted by the constrained case, where baseline hemodialysis growth was constrained to historic levels. In this scenario, ICERs for the combined interventions were considerably higher £15 092 (+11 246).

The validity of these findings was tested by comparison to external sources where available:

- The year 1 baseline burden of CKD estimated by the model was tested by comparison with a 2012 study which estimated the UK economic burden of CKD in 2010 at £1.45 billion.^6^ The like-for-like figure (NHS direct costs related to diagnosed patients only) from the model underpinning this study was £3.3 billion in 2023. This suggests a 6.5% compound annual growth rate in the burden of CKD in nominal terms or adjusting for UK inflation of 3.0% over the period, 3.5% annual growth in spend in real terms.^35^ This is in line with reported real-terms growth in overall healthcare expenditure of 3.6% for this period.^36^
- The ICER projected for intervention 3, SGLT-2 inhibitor uptake, also appears reasonable based on face validity. The model projected an ICER of £4287, while the NICE assessment of dapagliflozin reported an ICER of “around £6000 per QALY gained” for the 2 subgroups for which the product was recommended.

The study recognizes several limitations, including potential biases from combining data across various studies and not accounting for current NHS capacity constraints. Projections suggest a significant rise in CKD patients requiring dialysis or transplants over the next 10 years, potentially outpacing historical growth trends.

The results of this study should be understood in the context of the study’s limitations. First, the study scope was broad in that it sought to understand the impact of multiple interventions across all CKD patients. A consequence of the broad scope is that inputs have been sourced from multiple studies with overlapping, but not the same, patient populations from both clinical trials and registry studies ranging from 2001 to 2022, including potential biases from combining data across various studies. Moreover, the Markov model does not consider current capacity constraints faced by the UK NHS and changes to CKD treatment patterns. The model, using historical transition probabilities, predicts a significant rise (+300%) in CKD patients on dialysis or receiving kidney transplants over the next 10 years. This outpaces historical compound annual growth of both these treatments, which has hovered around 6% from 2015 to 2021.^13^

Future research should incorporate real-world treatment patterns and disease progression to refine model predictions and characterize the variance and increase the generalizability of the findings through a probabilistic sensitivity analysis.

## CONCLUSION

This cost-utility analysis underscores the need for the NHS to adopt a multifaceted approach to CKD management, integrating early diagnosis and innovative treatments to improve patient outcomes and reduce healthcare costs.

Among the 4 interventions analyzed, pre-emptive transplantation emerged as the most cost-effective option: resulting in reduced overall total cost of care. Greater use of SGLT-2 inhibitors led to the greatest QALY gains within the UK population overall.

While there is a body of clinical and real-world evidence which supports greater utilization of ACE-i/ARBs and SGLT-2 inhibitors, more evidence is needed on the effectiveness and costs of earlier diagnosis programs and programs that increase pre-emptive kidney transplantation, along with further research to evaluate effective ways of implementing these interventions in a real-world setting.

This research demonstrates the significance of progression to hemodialysis as a driver of cost and poor quality of life, and that a combination of interventions across the CKD disease pathway can prevent future CKD disease burden in the UK population.

## Supplementary Material

Online Supplementary Material
