# Supplementary material for: Economic Evaluation of Population-Level Chronic Kidney Disease Interventions in the UK National Health Service
Source: J Health Econ Outcomes Res. 2025 Apr 30;12(1):184–90. doi: 10.36469/001c.134075 (PMC12047453; doi:10.36469/001c.134075)
Supplement: Online Supplementary Material [file jheor_2025_12_1_134075_281051.pdf]

Journal of Health Economics and Outcomes Research

Online Supplementary Material

Economic Evaluation of Population-Level Chronic Kidney Disease Interventions in the UK National Health Service. *JHEOR*. 2025;12(1):184-190. doi:10.36469/jheor.2025.134075

Table S1: Key Parameters for Interventions

Table S2: Base Case Baseline Transition Probabilities (No Interventions Applied)

Table S3: Base Case Transition Probabilities With All 4 Interventions Applied

Table S4: Constrained Case Baseline Transition Probabilities (No Interventions Applied)

Table S5: Constrained Case Transition Probabilities With All 4 Interventions Applied

Table S6: Initial Distribution of Patients

Table S7: Calculation and Indexing Costs to 2022

This supplementary material has been provided by the authors to give readers additional information about their work.

This is an open-access article distributed under the terms of the Creative Commons Attribution 4.0 International License (CCBY-4.0). View this license's legal deed at http://creativecommons.org/licenses/by/4.0 and legal code at http://creativecommons.org/licenses/by/4.0/legalcode for more information.

Table S1. Key Parameters for Interventions

**Intervention**			**Source**
Intervention 1	Proportion of population who are undiagnosed (uncoded)	54%	Mollokia (2020)
	Percentage of adults with CKD who are BAME	15%	Source: UKKA report (2020-2021)
	Proportion of previously undiagnosed BAME patients in CKD stages 1, 2 and 3 who are diagnosed as a result of the intervention	25%	Assumption
	Cost of outreach program (excluding cost of testing)	£2 million/y	Assumption
	Annual cost of test per patient (uACR + GP appointment)	£55.00	NICE TA 775 (DAPA)
Intervention 2	Diagnosed CKD population not on ACE-i/ARB	29.79%	NICE TA 775 (DAPA)
	Relative risk reduction in CKD progression	0.28	Brenner et al (2001)
	Quarterly cost (ACEi/ARBs + monitoring and testing)	£19.06	NICE TA 775 (DAPA)
Intervention 3: Narrow population)	Relative risk reduction of heart failure hospitalization (patients with T2D)	0.7	Wheeler et al (2022)
	Relative risk reduction of heart failure hospitalization (patients without T2D)	0.79	Wheeler et al (2022)
	Relative risk reduction of ESKD (patients with T2D)	0.64	Wheeler et al (2022)
	Relative risk reduction of ESKD (patients without T2D)	0.50	Wheeler et al (2022)
	Annual estimated cost of SGLT-2i + 1 additional GP monitoring appointment	£507.00	NICE TA 775 (DAPA)
	Patients with CKD and no T2D eligible for SGLT-2i (% of total CKD population)	2.55%	NICE TA 775 (DAPA)
	Patients with CKD and T2D eligible for SGLT-2i (% of total CKD population)	16.25%	NICE TA 775 (DAPA)
	Total patients eligible for SGLT-2i (% of total CKD population)	18.80%	NICE TA 775 (DAPA)
Intervention 4: Pre-emptive transplant	Current % of transplants that are pre-emptive	18%	Calculated based on NHS Blood and Transplant Service. Annual Report on Kidney Transplantation (2022)
	Increase in pre-emptive transplants (%)	100%	Assumption
	Fixed cost of outreach (annual)	£149 000	Assumption

Abbreviations: ACEi, angiotensin-converting enzyme inhibitor; ARBs, angiotensin II receptor blockers; BAME; Black, Asian, and Minority Ethnic; CKD, chronic kidney disease; ESKD, end-stage kidney disease; GP, general practitioner; NHS, National Health Service; SGLT-2i, sodium-glucose transport protein-2 inhibitor; T2D, type 2 diabetes; uACR, ***.

Key assumptions utilized to identify the population for each intervention, the cost of the intervention, and the impact of the intervention.

Table S2. Base Case Baseline Transition Probabilities (No Interventions Applied)

Transition to	Transition from (%)															
	No Kidney Disease	Undiagnosed CKD				Diagnosed CKD					Transplantation		CVD		Dialysis	Death
		Stage 1	Stage 2	Stage 3	Stage 4	Stage 1	Stage 2	Stage 3	Stage 4	Stage 5	Acute	Post	Acute	Post		
No kidney disease	99.6%															
Undiagnosed CKD																
Stage 1	0.13%[a]	80.2%														
Stage 2		0.6%[b,i]	75.8%													
Stage 3			5.0%[b,i]	79.3%												
Stage 4				1.3%[b,i]	78.4%											
Diagnosed CKD																
Stage 1		18.6%[i]				98.8%										
Stage 2			18.6%[i]			0.6%	94.6%									
Stage 3				18.6%[i]			4.7%[b]	97.9%	1.7%[b]	0.1%[b]						
Stage 4					18.6%[i]			1.24%[b]	95.2%	1.3%[b]						
Stage 5				0.1%[b,i]	1.5%[b,i]			0.1%[b]	1.54%[b]	87.5%						
Transplantation																
Acute										0.8%[b]					1.5%[b]	
Post											100%	98.3%				
CVD																
Acute		0.4%[c]	0.4%[c]	0.4%[c]	0.6%[c]	0.4%[c]	0.4%[c]	0.37%[c]	0.62%[c]	0.8%[c]		1.0%[h]		2.7%[g]		
Post													100%	94.3%		
Dialysis										7.9%[b]		0.3%[b]			96.5%	
Death	0.3%[e,f]	0.3%[e,f]	0.3%[e,f]	0.4%[e,f]	0.9%[e,f]	0.3%[e,f]	0.3%[e,f]	0.4%[e,f]	0.9%[e,f]	1.7%[e,f]		0.4%[b]		3.0%[e,f]	1.9%[e,f]	100%

Abbreviations: CKD, chronic kidney disease; CVD, cardiovascular disease.
Quarterly transition probabilities were utilized to govern the flow of patients between states, including from the at-risk state (no CKD).
[a]Jonsson et al (2022).
[b]Nuijten et al (2012).
[c]Currie et al (2019).
[d]NICE (2022).
[e]Go et al (2004).
[f]ONS (2022).
[g]Schlackow et al (2017).
[h]Gill et al (2005).
[i]Hirst et al (2020).

Table S3. Base Case Transition Probabilities With All 4 Interventions Applied

**Transition to**	**Transition from No Kidney Disease (%)**															
No kidney disease	99.6%															
Undiagnosed CKD																
Stage 1	0.13%[a]	78.2%														
Stage 2		0.6%[b,i]	73.7%													
Stage 3			5.0%[b,i]	77.3%												
Stage 4				1.3%[b,i]	78.4%											
Diagnosed CKD																
Stage 1		20.6%[i]				98.8%										
Stage 2			20.6%[i]			0.6%	94.6%									
Stage 3				20.6%[i]			4.7%[b]	98.0%	1.7%[b]	0.1%[b]						
Stage 4					18.6%[i]			1.21%[b]	95.3%	1.3%[b]						
Stage 5				0.1%[b],i	1.5%[b,i]			0.1%[b]	1.49%[b]	87.4%						
Transplantation																
Acute										1.0%[b]					1.5%[b]	
Post											100%	98.3%				
CVD																
Acute		0.4%[c]	0.4%[c]	0.4%[c]	0.6%[c]	0.4%[c]	0.4%[c]	0.36%[c]	0.60%[c]	0.8%[c]		1.0%[h]		2.7%g		
Post													100%	94.3%		
Dialysis										7.9%[b]		0.3%[b]			96.5%	
Death	0.3%[e,f]	0.3%[e,f]	0.3%[e,f]	0.4 [e,f]	0.9%[e,f]	0.3%[e,f]	0.3%[e,f]	0.4%[e,f]	0.9%[e,f]	1.7%[e,f]		0.4%[b]		3.0%[e,f]	1.9%[e,f]	100%

Abbreviations: CKD, chronic kidney disease; CVD, cardiovascular disease.
Quarterly transition probabilities after adjustment to reflect the impact of interventions on the base case baseline. Highlighted cells are those which have been adjusted compared with **Table S2**.
[a]Jonsson et al (2022).
[b]Nuijten et al (2012).
[c]Currie et al (2019).
[d]NICE (2022).
[e]Go et al (2004).
[f]ONS (2022).
[g]Schlackow et al (2017).
[h]Gill et al (2005).
[i]Hirst et al (2020).

Table S4. Constrained Case Baseline Transition Probabilities (No Interventions Applied)

Transition to	Transition from (%)															
	No Kidney Disease	Undiagnosed CKD				Diagnosed CKD					Transplantation		CVD		Dialysis	Death
		Stage 1	Stage 2	Stage 3	Stage 4	Stage 1	Stage 2	Stage 3	Stage 4	Stage 5	Acute	Post	Acute	Post		
No kidney disease	99.6%															
Undiagnosed CKD																
Stage 1	0.13%[a]	80.2%														
Stage 2		0.6%[b,i]	75.8%													
Stage 3			5.0%[b,i]	79.3%												
Stage 4				1.3%[b,i]	78.4%											
Diagnosed CKD																
Stage 1		18.6%[i]				98.8%										
Stage 2			18.6%[i]			0.6%	94.6%									
Stage 3				18.6%[i]			4.7%[b]	97.9%	1.7%[b]	0.1%[b]						
Stage 4					18.6%[i]			1.2%[b]	95.2%	1.3%[b]						
Stage 5				0.1%[b,i]	1.5%[b,i]			0.1%[b]	1.54%[b]	95.2%						
Transplantation																
Acute										0.18%[b]					1.5%[b]	
Post											100%	98.3%				
CVD																
Acute		0.4%[c]	0.4%[c]	0.4%[c]	0.6%[c]	0.4%[c]	0.4%[c]	0.37%[c]	0.62%[c]	0.8%[c]		1.0%h		2.7%[g]		
Post													100%	94.3%		
Dialysis										0.7%[b]		0.3%[b]			96.5%	
Death	0.3%[e,f]	0.3%[e,f]	0.3%[e,f]	0.4%[e,f]	0.9%[e,f]	0.3%[e,f]	0.3%[e,f]	0.4%[e,f]	0.9%[e,f]	1.7%[e,f]		0.4%[b]		3.0%[e,f]	1.9%[e,f]	100%

Abbreviations: CKD, chronic kidney disease; CVD, cardiovascular disease.

Quarterly transition probabilities after adjustment to reflect historic growth in dialysis and transplant to provide a scenario where capacity is constrained. Highlighted cells are those which have been adjusted compared with **Table S2**.

[a]Jonsson et al (2022).
[b]Nuijten et al (2012).
[c]Currie et al (2019).
[d]NICE (2022).
[e]Go et al (2004).
[f]ONS (2022).
[g]Schlackow et al (2017).
[h]Gill et al (2005).
[i]Hirst et al (2020).

Table S5. Constrained Case Transition Probabilities With All 4 Interventions Applied

Transition to	Transition from (%)															
	Undiagnosed CKD					Diagnosed CKD					No CKD	Transplant: Post	No CKD	CVD: Post	No CKD	Death
	No CKD	Stage 1	No CKD	Stage 3	No CKD	Stage 1	No CKD	Stage 3	No CKD	Stage 5						
No kidney disease	99.6%															
Undiagnosed CKD																
Stage 1	0.13%[a]	78.2%														
Stage 2		0.6%[b,i]	73.7%													
Stage 3			5.0%[b,i]	77.3%												
Stage 4				1.3%[b,i]	78.4%											
Diagnosed CKD																
Stage 1		20.6%[i]				98.8%										
Stage 2			20.6%[i]			0.6%	94.6%									
Stage 3				20.6%[i]			4.7%[b]	98.0%	1.7%[b]	0.1%[b]						
Stage 4					18.6%[i]			1.2%[b]	95.3%	1.3%[b]						
Stage 5				0.1%[b,i]	1.5%[b,i]			0.1%[b]	1.49%[b]	95.3%						
Transplantation																
Acute										0.22%[b]					1.5%[b]	
Post											100%	98.3%				
CVD																
Acute		0.4%[c]	0.4%[c]	0.4%[c]	0.6%[c]	0.4%[c]	0.4%[c]	0.36%[c]	0.60%[c]	0.8%[c]		1.0%[h]		2.7%[g]		
Post													100%	94.3%		
Dialysis										0.7%[b]		0.3%[b]			96.5%	
Death	0.3%[e,f]	0.3%[e,f]	0.3%[e,f]	0.4%[e,f]	0.9%[e,f]	0.3%[e,f]	0.3%[e,f]	0.4%[e,f]	0.9%[e,f]	1.7%[e,f]		0.4%[b]		3.0%[e,f]	1.9%[e,f]	100%

Abbreviations: CKD, chronic kidney disease; CVD, cardiovascular disease.
Quarterly transition probabilities after adjustment to reflect the impact of interventions on the constrained scenario. Highlighted cells are those which have been adjusted compared with **Table S4**.
[a]Jonsson et al (2022).
[b]Nuijten et al (2012).
[c]Currie et al (2019).
[d]NICE (2022).
[e]Go et al (2004).
[f]ONS (2022).
[g]Schlackow et al (2017).
[h]Gill et al (2005).
[i]Hirst et al (2020).

Table S6. Initial Distribution of Patients

Health State	Proportion of Patients in State at t = 0	Reference
No kidney disease	86.12%[23,30]	Calculated
Undiagnosed CKD stage 1	1.76%[23,30]	
Undiagnosed CKD stage 2	1.62%[23,30]	
Undiagnosed CKD stage 3	2.56%[23,30]	
Undiagnosed CKD stage 4	0.08%[23,30]	
Diagnosed CKD stage 1	2.23%[23,30]	
Diagnosed CKD stage 2	2.06%[23,30]	
Diagnosed CKD stage 3	3.25%[23,30]	
Diagnosed CKD stage 4	0.11%[23,30]	
Diagnosed CKD stage 5	0.08%[23,30]	
Transplant (acute)	0.00%	Assumption
Transplant (post-acute)	0.07%[13]	UKKA (2021)
Dialysis	0.06%[13]	
CVD (acute)	0.00%	Assumption
CVD (post-acute)	0.00%	

Abbreviations: CKD, chronic kidney disease; CVD, cardiovascular disease.
At t = 0, patients were distributed across disease states based on the best available data on real-world prevalence and distribution of CKD in the United Kingdom.

Table S7. Calculation and Indexing Costs to 2022

Disease State	Annual Costs (£)				Quarterly Costs (2022) (£)
	Hospital Costs[a]	Primary Care Costs (Assumption)	Total Cost (2015)	Annual Costs Inflated (2022)	
Undiagnosed CKD G3	403	60	463	548.53	137.1332
Undiagnosed CKD G4	393	60	453	537.21	134.3032
Diagnosed CKD G3	403	60	463	548.53	137.1332
Diagnosed CKD G4	393	60	453	537.21	134.3032
Diagnosed CKD G5	525	60	585	694.45	173.6132
CVD (acute)	4350		4350	5153.60	5153.60
CVD (post-acute)	738		738	874.34	218.58

Abbreviations: CKD, chronic kidney disease; CVD, cardiovascular disease.
Annual costs taken from Kent et al (2015) (**Table 4**) were adjusted to include an estimate of primary care costs (as the original values covered only hospital costs), inflated to 2022 levels based on the UK Consumer Price Index and divided by 4 to reflect the quarterly cycles of the model.
[a]Kent et al (2015) (**Table 4**).
